# γ-Aminobutyric Acid Transporter 2 Mediates the Hepatic Uptake of Guanidinoacetate, the Creatine Biosynthetic Precursor, in Rats

**DOI:** 10.1371/journal.pone.0032557

**Published:** 2012-02-27

**Authors:** Masanori Tachikawa, Saori Ikeda, Jun Fujinawa, Shirou Hirose, Shin-ichi Akanuma, Ken-ichi Hosoya

**Affiliations:** 1 Department of Pharmaceutics, Graduate School of Medicine and Pharmaceutical Sciences, University of Toyama, Toyama, Japan; 2 Division of Membrane Transport and Drug Targeting, Graduate School of Pharmaceutical Sciences, Tohoku University, Sendai, Japan; Kanazawa University, Japan

## Abstract

Guanidinoacetic acid (GAA) is the biosynthetic precursor of creatine which is involved in storage and transmission of phosphate-bound energy. Hepatocytes readily convert GAA to creatine, raising the possibility that the active uptake of GAA by hepatocytes is a regulatory factor. The purpose of this study is to investigate and identify the transporter responsible for GAA uptake by hepatocytes. The characteristics of [^14^C]GAA uptake by hepatocytes were elucidated using the *in vivo* liver uptake method, freshly isolated rat hepatocytes, an expression system of *Xenopus laevis* oocytes, gene knockdown, and an immunohistochemical technique. *In vivo* injection of [^14^C]GAA into the rat femoral vein and portal vein results in the rapid uptake of [^14^C]GAA by the liver. The uptake was markedly inhibited by γ-aminobutyric acid (GABA) and nipecotinic acid, an inhibitor of GABA transporters (GATs). The characteristics of Na^+^- and Cl^−^-dependent [^14^C]GAA uptake by freshly isolated rat hepatocytes were consistent with those of GAT2. The Km value of the GAA uptake (134 µM) was close to that of GAT2-mediated GAA transport (78.9 µM). GABA caused a marked inhibition with an IC_50_ value of 8.81 µM. The [^14^C]GAA uptake exhibited a significant reduction corresponding to the reduction in GAT2 protein expression. GAT2 was localized on the sinusoidal membrane of the hepatocytes predominantly in the periportal region. This distribution pattern was consistent with that of the creatine biosynthetic enzyme, S-adenosylmethionine∶guanidinoacetate *N*-methyltransferase. GAT2 makes a major contribution to the sinusoidal GAA uptake by periportal hepatocytes, thus regulating creatine biosynthesis in the liver.

## Introduction

Guanidinoacetic acid (GAA) is the biosynthetic precursor of creatine which plays an important role in storage and transmission of phosphate-bound energy in tissues with high energy demands [1]. The physiological importance of creatine biosynthesis has been demonstrated by an inherited deficiency of the creatine biosynthetic enzyme, S-adenosylmethionine∶guanidinoacetate *N*-methyltransferase (GAMT) [2]. Patients exhibit a severe reduction in creatine and the simultaneous accumulation of GAA in the plasma, and brain, thus causing mental retardation, delayed speech, and epilepsy [3].

The liver is the main organ responsible for creatine biosynthesis. Indeed, high levels of GAMT mRNA are found in this tissue in humans and mice [4] and a severe reduction in GAMT activity has been detected in the liver of patients with GAMT deficiency [5]. However, the hepatocytes are incapable of the full synthesis of creatine from arginine and glycine whereas they readily convert GAA to creatine [6]. It is thus conceivable that GAA needs to be delivered from the circulating blood to hepatocytes for creatine synthesis.

We have found that GAA transport is mediated by a creatine transporter (CRT/Solute carrier SLC6A8) [7] and a taurine transporter (TauT/SLC6A6) [8]. However, CRT mRNA is not expressed in the human liver [9]. TauT-knockout mice exhibit only a 30% reduction in the taurine content of hepatocytes [10]. These lines of evidence prompted us to hypothesize that another transporter makes a substantial contributor to GAA uptake by hepatocytes.

Hepatocyte heterogeneity has been proposed in terms of transport and enzymatic activities [11]. The autoradiographical pattern of the liver after [^3^H]glutamate bolus administration shows that glutamate is exclusively taken up by pericentral hepatocytes, independent of the perfusion direction [11]. This is consistent with the immunohistochemical distribution of glutamine synthetase [12] which converts glutamate to glutamine. The functional coupling of glutamate uptake and glutamine synthesis in the pericentral hepatocytes raises the possibility that the sinusoidal transport of GAA and creatine synthesis occur in the same compartment of a particular hepatocyte population.

The purpose of this study is to identify the transporter responsible for GAA uptake by hepatocytes using a combination of the *in vivo* liver uptake method, freshly isolated hepatocytes, an expression system of *Xenopus laevis* oocytes, and RNA interference. The localization of the transporter in the liver was determined by immunohistochemical analysis.

## Materials and Methods

### Animals

Adult male Wistar rats (260–280 g) were purchased from Japan SLC (Hamamatsu, Japan). Mature female *Xenopus laevis* were purchased from Kato-S-Science (Chiba, Japan) and maintained in a controlled environment. All experiments were approved by the Animal Care Committee, University of Toyama (Protocol# S2008PHA-1, A2011PHA-15, A2011PHA-16).

### Reagents

[1-^14^C]Guanidinoacetic acid ([^14^C]GAA, 55 mCi/mmol), [^3^H]water (18 mCi/mol), and [4-^14^C]creatine ([^14^C]creatine, 50 mCi/mmol) were obtained from Moravek Biochemicals (Brea, CA), Parkin-Elmer Life and Analytical Sciences (Boston, MA), and American Radiolabeled Chemicals (St. Louis, MO), respectively. All other chemicals were commercial products of analytical grade.

### *In vivo* blood-to-liver transport of [^14^C]GAA after its femoral vein administration

Rats were anesthetized with an intraperitoneal injection of pentobarbital (50 mg/kg body weight), and [^14^C]GAA (3 µCi per rat) dissolved in 400 µL extracellular fluid buffer containing (in mM) 122 NaCl, 25 NaHCO_3_, 3 KCl, 0.4 K_2_HPO_4_, 1.4 CaCl_2_, 1.2 MgCl_2_, 10 D-glucose, and 10 HEPES was injected into the femoral vein. Tissue sampling and determination of radioactivity were performed according to a previous report [13]. As an index of the tissue distribution of GAA, the apparent liver-to-plasma concentration ratio was used. This ratio (mL/g liver) was defined as the amount of [^14^C] per gram liver divided by that per milliliter plasma, calculated over the time-period of the experiment. The results were shown as the apparent liver-plasma concentration ratio (mL/g liver) versus time (min).

### *In vivo* blood-to-liver transport of [^14^C]GAA after its portal vein administration

Rats were anesthetized by intraperitoneal injection of pentobarbital (50 mg/kg body weight), and their rectal temperatures were maintained at 37°C using a hot plate. The hepatic artery was ligated, and 200 µL Ringer's HEPES buffer (pH 7.4) containing both 10 µCi [^14^C]GAA or [^14^C]creatine and 0.5 µCi [^3^H]water as a highly diffusible internal reference, was rapidly injected into the portal vein. Eighteen seconds after injection, the right major lobe was excised from the liver and solubilized in Soluen-350 (Parkin-Elmer Life Science). The radioactivities of ^3^H and ^14^C in the liver and the injection solutions were determined using a liquid scintillation spectrophotometer (LSC-5000, Aloka, Tokyo, Japan). LUI is defined in equation 1, and was determined using equation 2:
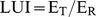
(1)

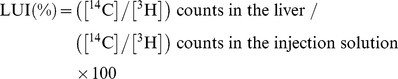
(2)Here, E_T_ and E_R_ are the fractions of [^14^C]GAA or [^14^C]creatine and [^3^H]water, respectively, extracted by the liver during a single pass. The value of E_T_ can be estimated when LUI and E_R_ are determined experimentally. Because the E_R_ value of [^3^H]water is reported to be 65±4% in rats [14], the following equation is valid:
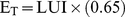
(3)The apparent fractional extractions consist of intracellular uptake, distribution to the interstitial space, and retention in the vascular space. Therefore, the extravascular extraction of [^14^C]GAA, which reflect only the intracellular uptake, was obtained as follows:
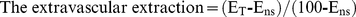
(4)Here, Ens represents the fractional extraction for distribution in the vascular and extracellular space. In this calculation, we used the reported E_ns_ value of 13±3% for rats [14].

### [^14^C]GAA uptake by freshly isolated hepatocytes

Rat hepatocytes were isolated by collagenase perfusion and Percoll isodensity centrifugation as described previously [15]. The hepatocytes were resuspended in Tyrode buffer containing (in mM) 137 NaCl, 2.7 KCl, 1.05 MgCl_2_, 1.8 CaCl_2_, 12 NaHCO_3_, 0.4 NaH_2_PO_4_, and 5.6 D-glucose. Uptake of [^14^C]GAA or [^14^C]creatine by the freshly isolated hepatocytes was examined according to the methods described previously [16]. In brief, after centrifugation and aspiration of the buffer, uptake was initiated by applying 100 µL Tyrode buffer containing [^14^C]GAA or [^14^C]creatine at 37°C in the presence or absence of inhibitors. Na^+^-free Tyrode buffer was prepared by replacement of NaCl, NaH_2_PO_4_ and NaHCO_3_ with equimolar N-methyl-D-glucamine, KH_2_PO_4_, and KHCO_3_, respectively. Cl^−^-free uptake buffer was prepared by replacement with equimolar gluconate. After a predetermined period, uptake was terminated by centrifugation of the solution. The cells were then solubilized in 1 N NaOH and subsequently neutralized with 1 N HCl. The cell-associated radioactivity and protein content were assayed by liquid scintillation spectrometry and detergent compatible protein assay (a DC protein assay kit, Bio-Rad, Hercules, CA) with bovine serum albumin as a standard.

### Kinetic analyses

The kinetic parameters for GAA uptake by freshly isolated rat hepatocytes were obtained from equation 5:
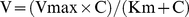
(5)where V is the uptake rate of GAA, C is the GAA concentration in the medium, Km is the Michaelis-Menten constant, and Vmax is the maximum uptake rate. To obtain kinetic parameters, the equation was fitted using the iterative non-linear least-squares regression analysis program, MULTI [17].

The median inhibitory concentration (IC_50_) value of GABA, taurine and creatine for [^14^C]GAA uptake by rat hepatocytes was estimated from equation 6, using MULTI.
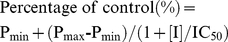
(6)where P_min_, P_max_, and [I] are the minimum percentage of control, the maximum percentage of control, and the concentration of inhibitor, respectively.

### RNA interference

RNA interference was performed using the BLOCK-iT Pol II miR RNAi expression kit (Invitrogen, Carlsbad, CA). Short hairpin RNAs (shRNAs) unrelated to and targeted to the GABA transporter (GAT) 2/SLC6A13 (Rmi603286, Invitrogen) were expressed in primary cultured rat hepatocytes by transient transfection using Lipofectamine 2000 reagent (Invitrogen) as previously reported [18].

### [^14^C]GAA and [^14^C]creatine uptake by rat GAT2-expressing *Xenopus laevis* oocytes

Using T7 RNA polymerase, capped cRNA was transcribed from *Not*I-linearized pGEM-HEN containing an open reading frame of rat GAT2 cDNA. Defolliculated oocytes were injected with 23 nL water or the capped cRNA (30–50 ng) and incubated at 18°C in freshly prepared standard oocyte saline solution containing 100 mM NaCl, 2 mM KCl, 1.8 mM CaCl_2_, 1 mM MgCl_2_, and 5 mM HEPES, 25 µg/mL gentamycin, 2.5 mM pyruvate and 1% bovine serum albumin, pH 7.5. The standard oocyte saline solution used to incubate the oocytes was replaced with fresh solution daily. Experiments were performed after incubation for 4 to 6 days. For the uptake study by *Xenopus laevis* oocytes, oocytes were preincubated with 500 µL ND96 solution containing (in mM) 96 NaCl, 2 KCl, 1.8 CaCl_2_, 1 MgCl_2_, and 5 HEPES for 20 min at 20°C before the uptake experiment. The uptake experiment was initiated by replacing the ND96 solution with 200 µL of the same solution containing [^14^C]GAA (45 µM) and [^14^C]creatine (45 µM). After incubation for a designated time at 20°C, the uptake was terminated by addition of ice-cold ND96 solution. Oocytes were then washed four times with ice-cold ND96 solution and solubilized in 5% sodium dodecyl sulfate solution, and the accumulated radioactivity was determined in a liquid scintillation counter (LSC-5000, Aloka).

### Antibody preparation

Polyclonal antibodies to organic anion transporting polypeptide 1a4 (oatp1a4/Slco1a4) and multidrug resistant protein 6 (MRP6/ABCC6) were raised against amino acid residues 625–661 of rat oatp1a4 (GenBank accession number: NP_571981) and 1468–1502 of rat MRP6 (GenBank accession number: NP_112275). The polypeptides were expressed as glutathione S-transferase (GST) fusion proteins using the pGEX4T-2 plasmid vector (GE Healthcare, Chalfont St. Giles, UK). The fusion protein was purified with glutathione-Sepharose 4B (GE Healthcare), emulsified with Freund's complete adjuvant (Difco, Detroit, MI), and injected subcutaneously into female Hartley guinea-pigs at intervals of 2 weeks. Two weeks after the sixth injection, affinity-purified antibodies were prepared, first using protein G-Sepharose (GE Healthcare) and then using antigen peptides coupled to cyanogens bromide-activated Sepharose 4B (GE Healthcare). For the preparation of affinity media, polypeptides free of GST were obtained by elution of the cleaved polypeptide after in-column thrombin digestion of fusion proteins bound to glutathione-Sepharose 4B.

### Immunoblotting

Under deep pentobarbital anesthesia (100 mg/kg body weight, i.p.), rat liver and heart were transcardially perfused with phosphate-buffered saline (PBS). The tissues were then homogenized using the nitrogen cavitation technique (800 psi, 15 min, 4°C) in buffer with the following composition (in mM): 10 HEPES, 1 EDTA, 1 EGTA, 320 sucrose, 1 phenylmethylsulfonyl fluoride, and a protease inhibitor cocktail (Sigma Aldrich, St. Louis, MO), pH 7.4. The homogenates were centrifuged at 10,000 g for 15 min. The supernatant fluids were further centrifuged at 100,000 g for 60 min to obtain a crude membrane fraction from the pellets. The protein concentration was determined using a DC protein assay kit (Bio-rad). Protein samples (50 µg per lane) were fractionated by sodium dodecyl sulfate (SDS)-polyacrylamide gel electrophoresis and electroblotted onto a nitrocellulose membrane. The blotted membrane was incubated with an affinity-purified antibody to GAT2 (Sigma Aldrich), oatp1a4, and MRP6 at 0.1–0.5 µg/mL in Tris-buffered saline (TBS; 25 mM Tris-HCl, pH 8.0 and 125 mM NaCl, pH 7.4) containing 0.1% Tween 20 and 4% skimmed milk for 16 h at 4°C, and visualized with an enhanced chemiluminescence kit (GE Healthcare).

### Immunohistochemistry

Under deep pentobarbital anesthesia (100 mg/kg body weight, i.p.), the liver of adult rats were obtained after transcardial fixation with 4% paraformaldehyde in 0.1 M sodium phosphate buffer (pH 7.4). The liver was immersed in 30% sucrose in 0.1 M sodium phosphate buffer. Frozen sections (40 µm in thickness) were prepared on a cryostat (CM1900; Leica, Nussloch, Germany). The sections were immunoreacted overnight with rabbit antibody to GAT2 (2 µg/mL, Sigma Aldrich), singly or in combination with guinea-pig oatp1a4 antibody (1 µg/mL), guinea-pig MRP6 antibody (1 µg/mL) and guinea-pig GAMT antibody (2 µg/mL, [19]). Subsequently, they were incubated with species-specific Alexa Fluor 488- (Invitrogen) and Cy3-conjugated secondary antibodies (Jackson ImmunoResearch, West Grove, PA) for 2 h. Photographs were taken using a confocal laser scanning microscope (TCS-SP5; Leica).

### Statistical analysis

All data except for kinetic parameters are presented as the mean±SEM. The kinetic parameters are presented as the mean±SD. An unpaired, two-tailed Student's *t*-test was used to determine the significance of differences between two group means. One-way analysis of variance followed by the modified Fisher's least-squares difference method was used to assess the statistical significance of differences among means of more than two groups.

## Results

### *In vivo* hepatic uptake of [^14^C]GAA and [^14^C]creatine

**Figure 1:**
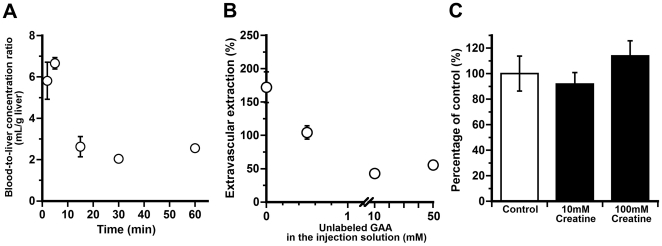
*In vivo* blood-to-liver transport of GAA. (A) [^14^C]GAA uptake by the liver after its intravenous administration. Each point represents the mean±SEM (n = 5–7). (B) Concentration-dependent uptake of GAA by rat liver after its injection into the portal vein. The extravascular extraction of [^14^C]GAA was plotted against the concentration of unlabeled GAA in the injection solution. Each point represents the mean±SEM (n = 3–4). (C) Simultaneous injection of unlabeled creatine had no effect on [^14^C]creatine uptake by the liver. Each column represents the mean±SEM (n = 5–6).

The liver-to-plasma concentration ratio of [^14^C]GAA rapidly increased up to 5 min after its intravenous administration (Figure 1A). The extravascular extraction of [^14^C]GAA after its bolus injection into the portal vein was estimated to be 182±1%, and this was reduced by increasing the unlabeled GAA concentration in the injection solution (Figure 1B). Table 1 shows the inhibition profile of *in vivo* hepatic uptake of [^14^C]GAA. GAA, GABA, and β-alanine produced a marked inhibition by more than 60% in each case at a concentration of 1 mM whereas taurine at the same concentration had no significant effect. GAA, GABA, β-guanidinopropionic acid, and nipecotic acid significantly inhibited the [^14^C]GAA uptake by more than 69% at a concentration of 10 mM. In contrast, taurine, creatine, creatinine, and L-alanine, each at a concentration of 10 mM, had a much weaker effect or no effect at all. The hepatic uptake of [^14^C]creatine was not inhibited by unlabeled creatine at concentrations of 10 mM and 100 mM (Figure 1C). These results indicate that the rapid accumulation of [^14^C]GAA in the liver is mediated by a carrier system preferring GABA over taurine and creatine.

**Table 1:** Effect of several compounds on *in vivo* [^14^C]GAA uptake by rat liver.

Inhibitors	Concentration of each inhibitor (mM)	% of control
Control		100±9
Guanidinoacetic acid (GAA)	1	39.4±2.9*
	10	29.4±2.8*
GABA	1	30.5±3.6*
	10	31.0±2.4*
β-Alanine	1	41.6±12.3*
Taurine	1	87.9±3.2
	10	63.9±8.7**
β-Guanidinopropionic acid	10	26.5±3.0*
Nipecotic acid	10	28.6±2.7*
Creatine	10	73.5±4.2**
Creatine and Taurine	10 and 10	35.7±7.6*
Creatinine	10	120±19
L-Alanine	10	121±21

Inhibitors dissolved in Ringer's HEPES buffer (pH 7.4) at the indicated concentrations were injected simultaneously with [^14^C]GAA (45 µM) into the rat portal vein. Each value represents the mean±SEM (n = 3–19).

*p<0.01,

**p<0.05, significantly different from the control.

### Kinetics and characteristics of [^14^C]GAA and [^14^C]creatine uptake by freshly isolated rat hepatocytes

**Figure 2:**
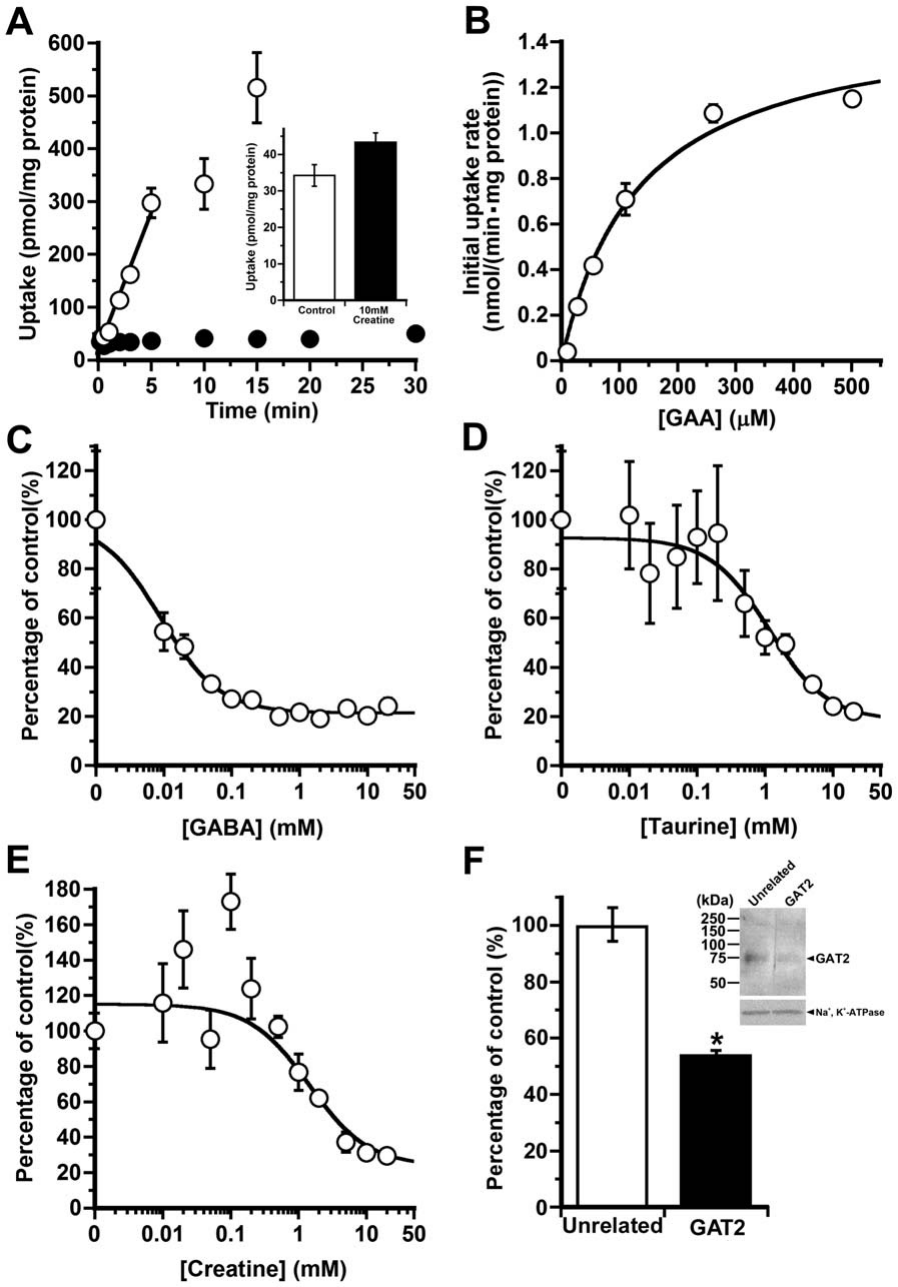
Characteristics of [^14^C]GAA uptake by freshly isolated rat hepatocytes. (A) Time-course of [^14^C]GAA (18 µM) uptake (open circle) and [^14^C]creatine (30 µM) uptake (closed circle) by hepatocytes. Inset graph shows no effect of unlabeled creatine (10 mM) on the [^14^C]creatine uptake at 2 min. Each point represents the mean±SEM (n = 3–4). (B) Concentration-dependence of GAA uptake by hepatocytes. The uptake was measured at the indicated concentration for 3 min. Each point represents the mean±SEM (n = 4). (C–E) Inhibitory effect of GABA (C), taurine (D), and creatine (E) on [^14^C]GAA uptake by freshly isolated rat hepatocytes. [^14^C]GAA (18 µM) uptake for 3 min at 37°C was measured in the presence and absence (control) of each compound at the designated concentrations. Each point represents the mean±SEM (n = 3–4). (F) Effect of treatment of shRNA, unrelated or targeted to GAT2, for 24 h on [^14^C]GAA (18 µM) uptake by primary cultures of rat hepatocytes at 37°C for 3 min. Immunoblotting (inset) with antibodies to GAT2 and Na^+^, K^+^-ATPase used as an internal standard shows the reduction of GAT2 protein expression in GAT2-silenced hepatocytes. Each column represents the mean±SEM (n = 4). *p<0.01, significantly different from the control.

[^14^C]GAA uptake by hepatocytes exhibited time-dependent increases up to 5 min (Figure 2A). In contrast, there was no time-dependent increase in [^14^C]creatine uptake by hepatocytes (Figure 2A). Unlabeled creatine, at a concentration of 10 mM, did not affect the [^14^C]creatine uptake (Figure 2A inset). The absence of either Na^+^ or Cl^−^ reduced the [^14^C]GAA uptake by 64.4% and 59.7%, respectively (Table 2). The [^14^C]GAA uptake took place in a concentration-dependent manner and consisted of a single saturable component (Figure 2B). The apparent Km and Vmax values of the saturable component were 134±27 µM and 1.53±0.12 nmol/(min·mg protein), respectively. Table 2 shows the inhibition profile of several compounds, each at a concentration of 2 mM, on the [^14^C]GAA uptake. GABA, β-alanine, GAA, β-guanidinopropionic acid, guanidinoethansulfonic acid and nipecotic acid produced a marked inhibition by more than 67%. In contrast, creatinine, betaine, guanidinosuccinic acid, L-arginine, L-alanine, and L-serine had much weaker effects. GABA, taurine, and creatine inhibited the [^14^C]GAA uptake in a concentration-dependent manner with an IC_50_ value of 8.81±1.33 µM, 1.13±0.30 mM, and 1.42±0.53 mM, respectively (Figure 2C–E). These results indicate that Na^+^- and Cl^−^-dependent GAA uptake by hepatocytes is mediated via a carrier system preferring GABA over taurine and creatine, most probably GAT. Among the GAT subtypes, GAT2 is predominantly expressed in rat liver [20]. A previous report has demonstrated that rat GAT2 was silenced by the expression of shRNA against rat GAT2 in a primary culture of rat hepatocytes [18]. [^14^C]GAA uptake was reduced by 46.4% in GAT2-silenced hepatocytes corresponding to the reduction in GAT2 protein (Figure 2F).

**Table 2:** Na^+^- and Cl^−^-dependence and inhibitory effect of several compounds on [^14^C]GAA uptake by freshly isolated rat hepatocytes.

Conditions/Inhibitors	% of control
Na^+^- and Cl^—^dependence	
Control	100±12
Na^+^-free	35.6±2.5*
Cl^—^free	40.3±1.6*
Inhibitory effect	
Control	100±4
Guanidinoacetic acid (GAA)	24.1±1.1*
GABA	17.1±1.6*
β-Alanine	28.5±5.5*
β-Guanidinopropionic acid	18.9±0.9*
Guanidinoethansulfonic acid	33.0±4.4*
Nipecotic acid	30.8±1.2*
Creatinine	75.3±8.9**
Guanidinosuccinic acid	71.2±5.1*
Betaine	64.4±8.7*
L-Arginine	74.9±11.1*
Glycine	54.4±11.2*
L-Alanine	59.6±7.8*
L-Serine	83.8±6.4

[^14^C]GAA (18 µM) uptake by hepetocytes was measured at 37°C for 3 min in the absence or presence (control) of Na^+^ and Cl^−^ or in the absence (control) or presence of inhibitors (2 mM). Each value represents the mean±SEM (n = 3–4).

*p<0.01,

**p<0.05, significantly different from the control.

### Characteristics of [^14^C]GAA uptake in rat GAT2-expressing Xenopus oocytes (GAT2/oocytes)

**Figure 3:**
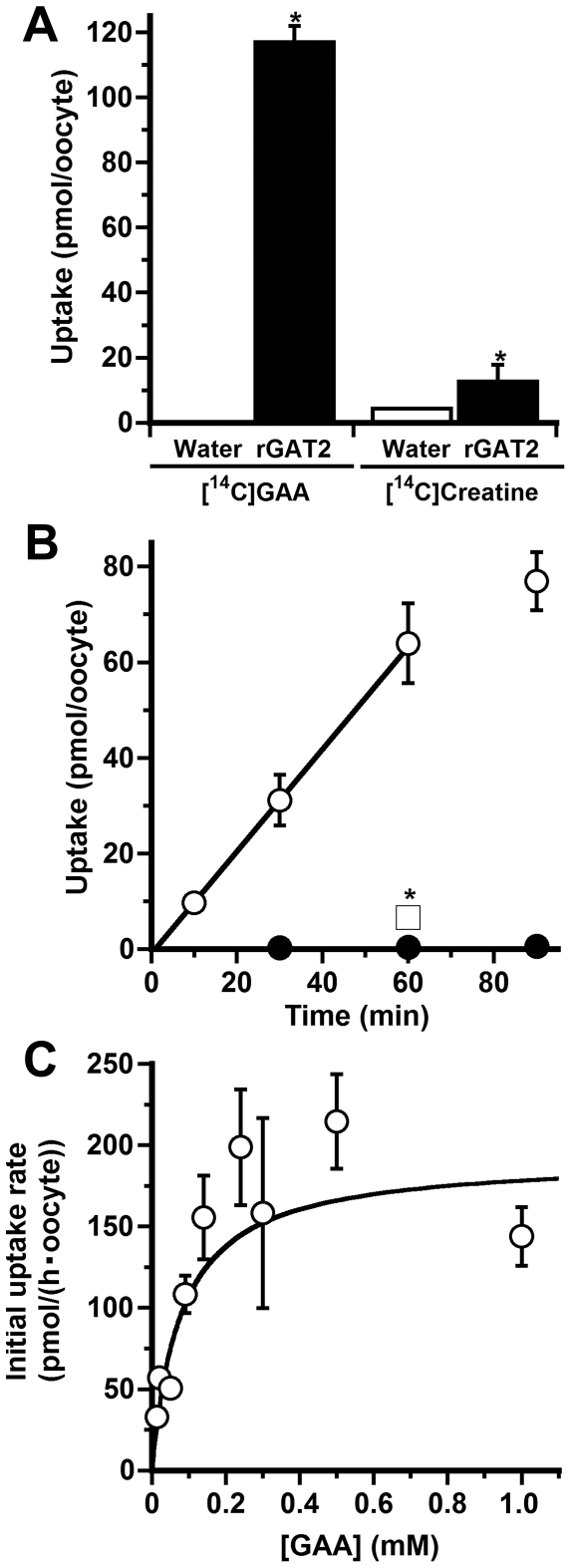
Characteristics of GAA transport in *Xenopus laevis* oocytes expressing GAT2 (GAT2/oocytes). (A) Uptake of [^14^C]GAA (45 µM), and [^14^C]creatine (45 µM) by oocytes injected with water (Water; open column) and GAT2 cRNA (GAT2; closed column) for 1 h at 20°C. Each column represents the mean±SEM (n = 8–14). *p<0.01, significantly different from the control. (B) Time-courses of [^14^C]GAA uptake (45 µM) by oocytes injected with water (closed circle) and GAT2 cRNA (open circle) at 20°C. The [^14^C]GAA uptake was inhibited by unlabeled GAA (10 mM; open square). Each point represents the mean±SEM (n = 9–15). *p<0.01, significantly different from the control. (C) Concentration-dependence of [^14^C]GAA uptake by GAT2/ooccytes at 20°C. The uptake was measured at the indicated concentration for 1 h. Each point represents the mean±SEM (n = 5–15).

[^14^C]GAA uptake by GAT2/oocytes was 433-fold greater than that by water-injected oocytes whereas [^14^C]creatine uptake by GAT2/oocytes was 28-fold higher than in water-injected oocytes (Figure 3A). The [^14^C]GAA uptake by GAT2/oocytes exhibited a time-dependent increase, and the uptake in these oocytes was several-fold higher than in water-injected oocytes (Figure 3B). The GAA uptake by GAT2/oocytes exhibited saturable kinetics with a Km of 78.9±26.4 µM and a Vmax of 193±32 pmol/(h·oocyte) (Figure 3C). The inhibitory effects of GAT substrates and inhibitors on the [^14^C]GAA uptake were further examined (Figure 3B and Table 3). GAA, GABA, β-alanine, γ-guanidinobutyric acid, and β-guanidinopropionic acid exhibited a marked inhibition of over 95%, each at a concentration of 5–10 mM. Nipecotic acid, taurine, guanidinoethansulfonic acid, and creatine inhibited the [^14^C]GAA uptake by more than 42%, each at a concentration of 5 mM. In contrast, creatinine, methlguanidine, guanidinosuccinic acid, betaine, L-alanine, homovanilic acid, and L-arginine did not produce any significant inhibition.

**Table 3:** Effect of several compounds on [^14^C]GAA uptake by *Xenopus laevis* oocytes expressing rat GAT2 (GAT2/oocytes).

Inhibitors	% of control
Control	100±7
GABA	1.74±0.44*
β-Alanine	3.54±0.64*
β-Guanidinopropionic acid	0.81±0.07*
γ-Guanidinobutyric acid	2.35±0.23*
Taurine	16.6±4.4*
Guanidinoethansulfonic acid	11.0±1.2*
Nipecotic acid	11.9±1.5*
Creatine	57.3±8.3**
Creatinine	147±18
Methlguanidine	131±17
Guanidinosuccinic acid	148±11
Betaine	96.7±7.4
L-Alanine	138±18
Homovanilic acid	85.3±7.0
L-Arginine	137±15

[^14^C]GAA (45 µM) uptake by GAT2/oocytes was measured at 20°C for 1 h in the absence (control) and presence of inhibitors (5 mM). Each value represents the mean±SEM (n = 10–15).

*p<0.01,

**p<0.05, significantly different from the control.

### Expression and localization of GAT2 in rat liver

**Figure 4:**
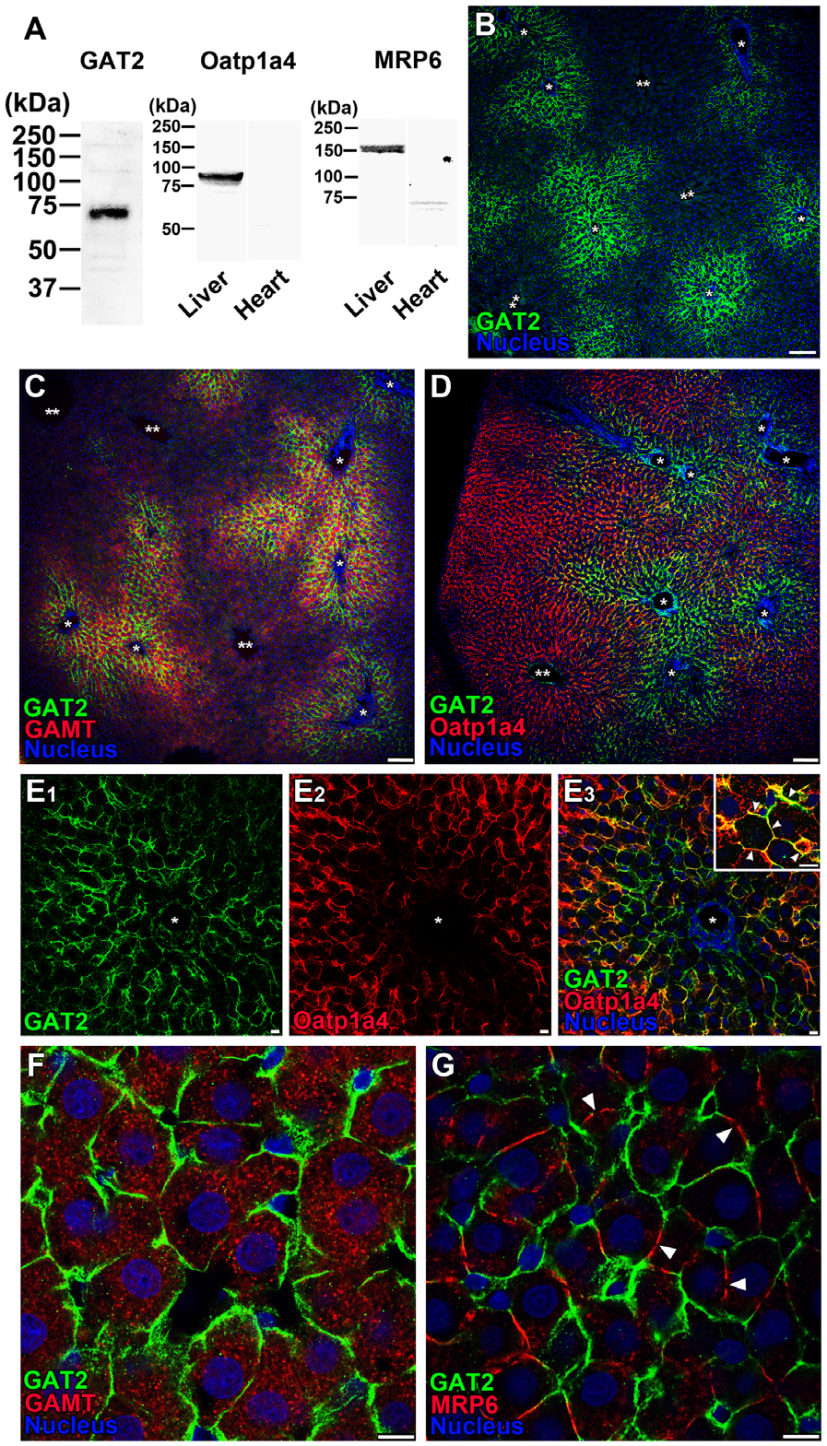
Expression and localization of GAT2 in rat liver. Red and green fluorescence is defined at the lower left corner of each panel. (A) Immunoblot of GAT2, oatp1a4, and MRP6 in rat liver and heart. Rat heart was used as negative control for oatp1a4 and MRP6 expression. The size of the marker proteins is indicated to the left. (B–C) Preferential distribution of GAT2 (B and C) and GAMT (C) in hepatocytes around the portal vein. (D–E) Distribution of GAT2 and oatp1a4 in the liver. E_3_ inset: Localization of GAT2 on the oatp1a4-positive sinusoidal membrane (arrowheads). (F) Plasma membrane localization of GAT2 in GAMT-positive hepatocytes. (G) Double immunofluorescence of GAT2 and MRP6, a marker of the lateral membrane of hepatocytes. Arrowheads indicate the bile canaliculus. Nuclei were stained with 4′,6-diamidino-2-phenylindole (DAPI, blue). In B–E, single (*) and double (**) asterisks indicate the portal spaces and the central vein, respectively. Scale bars: B–D, 100 µm; E–G, 10 µm.

Using immunoblotting with the crude membrane fraction from rat liver, the antibodies to GAT2, oatp1a4, and MRP6 recognized a single band at 70 kDa, 92 kDa, 178 kDa, respectively (Figure 4A). The size of each band detected was consistent with the previous results [21], [22]. On the other hand, no band was detected in rat heart used as negative control for oatp1a4 and MRP6 expression (Figure 4A). Therefore, the antibodies to GAT2, oatp1a4, and MRP6 were judged to be specific. The GAT2 antibody predominantly labeled hepatocytes in the periportal region rather than pericentral region (Figure 4B). The intense signals of GAMT were also distributed predominantly in the periportal region than in the pericentral region (Figure 4C). In contrast, the reverse was found for the distribution patterns of GAT2 and oatp1a4: oatp1a4 was predominantly distributed in the pericentral region (Figure 4D–E). The immunoreactivities of GAT2 were partially overlapped with those of oatp1a4 which is localized on the sinusoidal membrane in the liver (Figure 4E_3_ inset). The immunostaining of rat GAT2 was localized on the plasma membrane of hepatocytes which express GAMT (Figure 4F). Using double immunofluorescence for MRP6, a marker of the lateral membrane of hepatocytes, the GAT2 immunoreactivities did not overlap with those of MRP6 (Figure 4G). These results indicate that GAT2 is preferentially localized on the sinusoidal membrane of hepatocytes in the periportal region.

## Discussion

The present study demonstrates that GAT2 is responsible for the rapid uptake of GAA on the sinusoidal membrane of hepatocytes predominantly in the periportal region.

*In vivo* bolus injection of [^14^C]GAA into the rat femoral vein or portal vein results in its rapid extraction by the liver (Figure 1A and B). Considering that the GAA concentration in the central vein is lower than that in the hepatic artery and portal vein [6], the liver would be responsible for GAA clearance from the circulating blood. Since GABA and GAT inhibitors, such as β-guanidinopropionic acid and nipecotinic acid, produce a marked inhibition (Table 1), an isoform of GATs is most likely involved in the GAA uptake by the liver. Furthermore, the degree of inhibition by β-alanine is comparable with that by GAA and GABA, each at a concentration of 1 mM (Table 1), suggesting that GAA, GABA and β-alanine share a common transport system in the liver. Previous reports have demonstrated that GAT2 mediates the uptake of β-alanine and its analog α-fluoro-β-alanine by rat hepatocyets [23] and that α-fluoro-β-alanine rapidly accumulates in the rat liver after its intravenous administration [24]. It thus appears that the rapid uptake of [^14^C]GAA by the liver is also mediated by GAT2. In support of this notion, the characteristics of Na^+^- and Cl^−^-dependent [^14^C]GAA uptake by freshly isolated rat hepatocytes (Figure 2 and Table 2) support the hypothesis that GAT2 plays a major role in GAA uptake by hepatocytes, especially based on the following evidence. (i) The Km value (134 µM; Figure 2B) and the inhibition profile (Table 2) of the [^14^C]GAA uptake is in good agreement with those of GAT2-mediated GAA transport (Figure 3C and Table 3). (ii) GABA causes a marked reduction in the [^14^C]GAA uptake with the lowest IC_50_ value (Figure 2C). (iii) [^14^C]GAA uptake exhibits a significant reduction corresponding to the decrease in the protein expression of GAT2 (Figure 2F). The Na^+^-dependence (64.4%; Table 2) of the [^14^C]GAA uptake is almost identical to the degree of inhibition by 2 mM GABA (approximately 80%; Table 2). Therefore, GAT2 would make at least a 64.4% contribution to the total [^14^C]GAA uptake by hepatocytes.

The freshly isolated rat hepatocytes have much higher GAA uptake activity compared with creatine uptake (Figure 2A) whereas the liver concentration of creatine is 100-to-680-fold greater than that of GAA in humans and rats [25]. This confirms the belief that GAA is readily converted to creatine [6] as soon as GAA is taken up by hepatocytes. Immunohistochemical analysis reveals that GAT2 is localized on the sinusoidal membrane of hepatocytes predominantly in the periportal region (Figure 4). This distribution pattern is in good agreement with that of GAMT, the creatine synthetic enzyme (Figure 4). The functional coupling between GAT2 and GAMT suggests that the supplying pathway for GAA regulates creatine synthesis in periportal hepatocytes.

It has become evident that hepatocytes located at different distances from the portal spaces exhibit different transport characteristics. This includes the preferential uptake of glutamate [11] and taurocholate [26] by pericentral and periportal hepatocytes, respectively, after injection into the portal vein. However, only a limited number of studies have addressed the question as to whether this heterogeneity is merely due to the location of hepatocytes or to intrinsic differences between the cells. The present study provides evidence to support the hypothesis that GAT2 is responsible for the periportal region-predominant uptake of GAA by GAMT-expressing hepatocytes and supports the latter concept. This zonal arrangement for creatine synthesis in the liver may explain the discrepancy that although the GAA biosynthetic enzyme, L-arginine∶glycine amidinotransferase (AGAT), is expressed predominantly in pericentral hepatocytes [27], the hepatocytes are incapable of the full synthesis of creatine from arginine and glycine [6].

GAT2 mediates the hepatic uptake of GABA, β-alanine, and γ-butyrobetaine, a synthetic precursor of carnitine, with the Km values of 9–35.3 µM [18], [23]. Since the blood concentrations of GABA (0.13 µM; [28]), β-alanine (<0.5 µM; [29]), and γ-butyrobetaine (0.84–0.95 µM; [30]) are smaller than the Km value of GAT2-mediated transport for each substrate, GAT2 in the hepatocytes would not be fully saturated by endogenous substrates of GAT2 under physiological conditions. The GAA uptake by rat hepatocytes with a Km value of 134 µM (Figure 2) would operate at only a small fraction of its maximal activity at a plasma concentration of 3.73 µM [25]. However, such a transport system would be relevant for GAA with fluctuations in its plasma concentration. Creatine supplementation decreases the blood concentration of GAA due to the downregulation of AGAT activity in the kidney [6]. Since excess creatine does not alter GAMT activity in the liver [6], the decrease in GAT2-mediated GAA uptake by hepatocytes results in the reduction of creatine biosynthesis in the liver. Patients with GAMT deficiency exhibit a marked reduction in creatine and a simultaneous accumulation of GAA (12.9–20.7 µM; [31]) in the circulating blood. Although oral treatment with creatine increases its plasma concentration above the normal range (270–763 µM), the problem is that the GAA concentration in the plasma remains elevated [31]. The present study indicates that creatine affects GAA uptake by hepatocytes and GAT2-mediated GAA transport (Figures 2E and 3A, and Tables 2 and 3), implying that the hepatic clearance of GAA from the circulating blood is disturbed by increased levels of creatine. Therefore, monitoring the creatine concentration in the plasma will be beneficial to ensure that the plasma level of creatine will not exceed the normal range.

The liver does not express any isoforms of creatine kinases [32], [33] and a carrier system for creatine uptake (Figures 1C and 2). This implies that creatine produced in hepatocytes is released into the blood stream. Nevertheless, the creatine level in the liver remains higher than that in the blood [25], raising the possibility that the liver serves as the storage compartment for creatine. It is thus intriguing to pursue the mechanism(s) of the continuous release or stimuli-dependent release of creatine from hepatocytes.

In conclusion, GAT2 makes a major contribution to GAA uptake by periportal hepatocytes on the sinusoidal membrane. The present findings shed light on a regulatory system for creatine biosynthesis and a functional heterogeneity of hepatocytes.
